# Advances in the Development of Innovative Sensor Platforms for Field Analysis

**DOI:** 10.3390/mi11050491

**Published:** 2020-05-11

**Authors:** Silvia Rizzato, Angelo Leo, Anna Grazia Monteduro, Maria Serena Chiriacò, Elisabetta Primiceri, Fausto Sirsi, Angelo Milone, Giuseppe Maruccio

**Affiliations:** 1 Department of Mathematics and Physics “Ennio De Giorgi”, University of Salento, Omnics Research Group, Via per Monteroni, 73100 Lecce, Italy; annagrazia.monteduro@unisalento.it (A.G.M.); fausto.sirsi@unisalento.it (F.S.); angelo.milone@unisalento.it (A.M.); giuseppe.maruccio@unisalento.it (G.M.); 2 Institute of Nanotechnology, CNR-Nanotec, Omnics Research Group, Via per Monteroni, 73100 Lecce, Italy; mariaserena.chiriaco@nanotec.cnr.it (M.S.C.); elisabetta.primiceri@nanotec.cnr.it (E.P.)

**Keywords:** environmental monitoring, sensors, remote sensing

## Abstract

Sustainable growth, environmental preservation, and improvement of life quality are strategic fields of worldwide interest and cornerstones of international policies. Humanity health and prosperity are closely related to our present choices on sustainable development. The main sources of pollution concern industry, including mining, chemical companies, and refineries, wastewater treatment; and consumers themselves. In order to guide and evaluate the effects of environmental policies, diffuse monitoring campaigns and detailed (big) data analyses are needed. In this respect, the development and availability of innovative sensor platforms for field analysis and remote sensing are of crucial relevance. In this review, we provide an overview of the area, analyzing the major needs, available technologies, novel approaches, and perspectives. Among environmental pollutants that threaten the biosphere, we focus on inorganic and organic contaminants, which affect air and water quality. We describe the technologies for their assessment in the environment and then draw some conclusions and mention future perspectives opened by the integration of sensing technologies with robotics and the Internet of Things. Without the ambition to be exhaustive in such a rapidly growing field, this review is intended as a support for researchers and stakeholders looking for current, state-of-the-art, and key enabling technologies for environmental monitoring.

## 1. Introduction

Sustainable growth, environmental preservation, and improvement of life quality are strategic fields of worldwide interest and cornerstones of international policies. As pointed out in the latest United Nations Global Environment Outlook, GEO-6 [1], humanity health and prosperity are closely related to our present choices on sustainable development. It is estimated that a quarter of premature deaths and illnesses in the world are related to man-made pollution, which has seriously compromised a large part of resources and conditions essential for human life and health, namely air, water, soil, and food. As examples, the scarcity or absence of drinking water causes 1.4 million deaths per year, while chemical agents as pesticides [2], industry waste effluent [3], and vehicle exhausts [4] potentially cause even multi-generational effects. The main sources of pollution concern industry, including mining, chemical companies, and refineries; wastewater treatment; and consumers themselves.

In order to guide and evaluate the effects of environmental policies, diffuse monitoring campaigns and detailed (big) data analyses are needed. In this respect, the development and availability of innovative sensor platforms for field analysis and remote sensing are of crucial relevance. In this review, we provide a concise overview of this important area, analyzing the main environmental pollutants, major needs, available technologies, novel approaches, and future perspectives. Among environmental pollutants or xenobiotic compounds that threaten the biosphere, we will consider the main sources of contamination, inorganic and organic, that affect air and water quality. Specifically, we focus on particulate matter in air and microplastics in water, which share size ranges and some sensing requirements; heavy metal ions in water; small molecules as combustion products; and hazardous gases in air or pesticides/biocides in water. Hydrocarbons in oil form are not reviewed because they are recently considered more from the bioremediation point of view. On the other hand, noise pollution is also discussed as a physical more than particle/chemical source. For each contaminant, we summarize the fundamental characteristics, health effects, and most used methods of detection, without going into details of the techniques, but describing problems, challenges, and limits of detections. In order to focus the reader on the general context, in terms of methodology, for each category, we mention a few examples from the literature chosen on the basis of impact and relevance of the selected publications according to the received citations, the journal impact factor, and their novelty (for example, the Web of Science Database was employed, with the following search terms: “portable sensors combined with environmental monitoring, particulate matter, microplastics, heavy metals, pesticides/biocides, environmental noise pollution, air quality guidelines, water quality guidelines”; we used both seminal publications with high impact on the field and recent articles published in the last five years). Without the ambition to be exhaustive in such a rapidly growing field, this review is indeed intended as a support for researchers and stakeholders looking for current, state-of-the-art, and key enabling technologies for environmental monitoring.

In the following sections, we start discussing particulate matter and monitoring of microplastics in Section 2 and Section 3, and then we describe technologies for assessment of heavy metals and small molecules pollution (combustion products and hazardous gases as well as pesticides/biocides) in Section 4, Section 5, and Section 6. Successively, we review noise pollution assessment in Section 7, while in Section 8, for each pollutant, we summarize the status in terms of guideline values, known health effects, sources, and limits of detection before discussing the perspectives opened by the integration of sensing technologies with robotics and the Internet of Things. Finally, in Section 9, we draw some conclusions.

## 2. Particulate Matter

A major risk for human health is exposure to air pollution owing to ambient particulate matter (PM). The term PM refers to a complex and heterogeneous ensemble made of different components with various physical characteristics. The constituents can be classified as primary and secondary particles, based on the sources that produce them, natural and anthropic sources (from volcanic eruptions to industrial processes), respectively, on one side [5], and nucleation, coagulation, and condensation of molecules present in the gas phase on the other one [6]. In terms of PM chemical composition, we can distinguish three main classes: inorganic ions, carbonaceous fraction, and crustal material; another fourth part is related to the presence of water. The size of particles composing PM varies from a few nanometers to tens of micrometers, but the literature reports a net distinction between particles smaller than 2.5 µm, particles between 2.5 µm and 10 µm, and particles up to 100 µm; if the first ones penetrate the alveoli and terminal bronchioles, the second ones mainly deposit in the primary bronchi, while the latter ones are withheld by nasopharynx [5]. To have an idea of the impact and threat, it was estimated that 2016 exposure to particle matter with diameters below 2.5 µm (PM_2.5_) reduced average global life expectancy at birth by one year, with peaks of almost two years in Asia and Africa [7]. Among all the PM, the ultrafine particles (with diameter less than 0.1 µm (PM_0.1_)) represent the worst hazard for health as they are able to overstate the air–blood barrier near the lungs. The induced adverse health outcomes result in strokes, lung cancer, chronic obstructive pulmonary disease, respiratory infections, and ischemic heart disease.

Airborne particulate detection is traditionally carried out using optical and gravimetric approaches such as ellipsometry, light scattering-based instruments, and tapered element oscillating microbalance spectrometers [8,9,10]. All these techniques have demonstrated high sensitivity and reliability, but require long time analysis as well as heavy and expensive instrumentation, and are thus not usually available as on-field tools. Recently, however, different approaches have been developed to decrease the volume and cost of air particulate monitoring systems.

**Figure 1:**
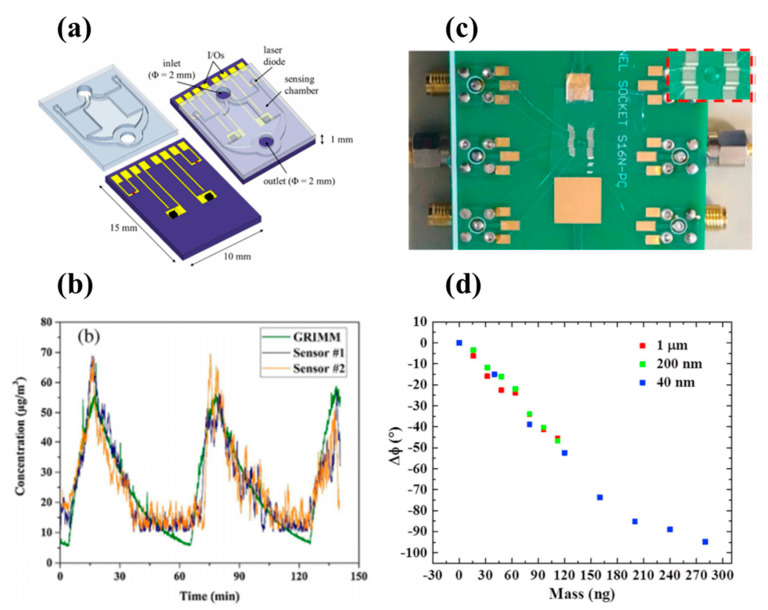
(**a**) Schematic of a light scattering−based sensor and (**b**) its relative representative reading (reproduced from [12]). (**c**) Surface acoustic wave (SAW) delay lines connected to a printed circuit board and (**d**) relative phase shifts as a function of mass for particles of different diameter size (reproduced with permission from [14]).

Light scattering is one of the most applied approaches in the realization of portable detectors for the measurement of size and concentration of airborne particulate matter. For example, in [11], the authors reported a miniaturized device exploiting a system of three Fresnel ring lenses for collecting scattered light on two solid angle intervals. Particles coming into a measuring chamber cross a laser beam and scatter light, which is collimated by a first Fresnel lens and then focused onto two avalanche photodiodes by two separate Fresnel ring lenses. This innovative system enhancing the collected signal allows to detect particle of size down to 150 nm. A simpler and low cost miniaturized PM sensor based on the light scattering method was achieved in [12] (Figure 1a,b), where the authors mounted a low power laser source and a photodiode at the diagonally opposite corners of a small chamber of sizes 15 × 10 × 1 mm^3^ consisting of two stacked silicon submounts. Both laboratory and field testings carried out on this sensor showed a good accuracy of less than 10 μg/m^3^ and a rapid response to particle concentration variations. 

Acoustic wave-based devices, such as quartz microbalance crystals (QCM) and surface acoustic wave (SAW) devices, have been developed to replace the traditional gravimetric methods, in which the particle mass concentration was determined by weighing the air filters before and after the sampling period. In order to overcome this disadvantage and realize portable devices able to monitor the exposure to aerosols in real time, it was proposed to collect the particles on the surface of the electromechanical sensors (QCM or SAW) and to measure the accumulated mass as a shift of device resonance frequency. QCM operates by using bulk acoustic waves excited through the application of an AC voltage to the electrodes patterned on both sides of a piezoelectric substrate (i.e., AT-cut quartz), whereas SAW transducers consist of interdigital electrodes whose periodicity matches the SAW wavelength at a specific frequency. SAW-based sensors possess higher sensitivity toward mass changes than QCM as the energy is confined on the surface region rather than in the bulk and the working frequencies are at least an order of magnitude higher than QCM (up to GHz compared with 5−20 MHz of QCM).

In [13], a QCM sensor integrated in a three-dimensional printed virtual impactor was developed for the separation and detection of particulate matter. The authors demonstrated that this system was able to separate silicon dioxide particles with a diameter smaller than 2.5 μm from the inlet particle flow (having a diameter in the range of 0.5–8 μm) and to detect them with a good sensitivity of 0.274 Hz/ng.

Concerning the detection of sub-micrometer particles, in [14], surface acoustic wave (SAW) delay lines were comparatively investigated with electrochemical impedance spectroscopy (EIS)-based devices. Specifically, both transduction methods were able to detect the presence of particulate matter of size down to 200 nm, which is considered the most harmful as it is able to penetrate deep into the lungs and blood streams. The SAW-based sensor (Figure 1c–d) was more performant in terms of sensitivity and detection of nanoscale particles (down to 40 nm), although the EIS approach had a good sensitivity considering also its even lower cost.

In [15], Thomas and collaborators investigated a two-port SAW resonator for detection of micron and submicron sized particles, showing a mass sensitivity depending on the particle diameter (with respect to the acoustic wavelength). In particular, this parameter is higher for particles having size comparable or smaller than the acoustic penetration depth. Moreover, the authors demonstrated the ability of the SAW resonator to detect masses below 1 ng with a higher sensitivity of 275 Hz/ng.

## 3. Microplastics

The term microplastics refers to plastic particles of size in the range of 0.001–5 mm, resulting from the fragmentation of larger plastic objects (owing to oxidation, mechanical forces, UV radiation) or intentionally designed to be small for use in beauty and health products. Because of their physical dimension, microplastics can be accidentally ingested by aquatic life and birds, and thus get accumulated in the food chain, compromising ecosystems and human health. Microplastics can be collected using plankton nets of different mesh size [16] for analysis in a laboratory. The different properties of these small materials, such as shape, size distribution, and light appearance (transparency and translucent), as well as the refractive index close to water values, make their detection in practical field condition difficult.

Traditionally, more than one analytical method has been employed for the identification of microplastics. The first step consists of the physical characterization of potential plastic through a visual inspection [17]. Specifically, whereas larger plastics (2–5 mm) can be easily detected with naked eye observation, plastic objects of hundred micrometers in size are generally identified by means of magnified images obtained using optical microscopy, which can give information about surface texture and structure. Indeed, a certain number of selection criteria, concerning geometry, color, and degradation stage, are proposed to aid the positive identification of plastic particles through the visual method [17]. However, this approach is not recommended for the detection of microplastics particles <500 μm (especially if transparent and without a specific shape), as it does not allow to discriminate, without doubt, plastic from other organic materials, and thus may lead with high probability to a false identification of plastic-like particle.

Scanning electron microscopy (SEM) is able to solve this problem, providing high-resolution images of small plastic particles. This technique is generally complemented with energy-dispersive X-ray spectroscopy (EDS), which provides information about the elemental composition of the analyzed objects [18]. Although the combination of SEM–EDS works well in the differentiation of microplastics from other small samples, it is not appropriate for studying a large number of samples as it needs a lot of time and effort for sample preparation and investigation.

When the size of microplastics is lower than 1 mm, the microscopy analysis is supported by a second step of chemical characterization for the confirmation of the material type under examination. Plastic are long chains of polymers mainly composed of carbon, hydrogen, and oxygen. The polymer composition of microplastics may be identified by means of spectroscopic techniques such as Fourier transform infrared (FTIR) spectroscopy, Raman scattering, or thermal analysis as pyrolysis gas chromatography-mass spectrometry (py-GC-MS).

Py-GC-MS spectrometry enables the determination of microplastics polymers by analyzing gases that are thermally decomposed starting from polymers, and by comparing their characteristic pyrogram with reference curves of known polymers [19]. Despite that this technique requires small amounts of sample, making possible its application for trace analysis, it has the disadvantage of being a destructive method and does not allow further analysis of microplastics samples.

FTIR spectroscopy is one of the most used techniques for chemical analysis of environmental samples, able to reveal polymer composition, and providing an IR spectrum that contains individual peaks corresponding to specific bonds in the chemical compound. IR spectra give information about microplastics’ polymer types and its abundance, as well as the physiochemical weathering process, by analyzing the relative composition of oxygenated bonds [17]. When the plastic particle size decreases below 10 µm, IR spectra lose accuracy, making plastic identification difficult.

Raman spectroscopy is another recommended technique for studying the chemical structure of suspected plastics, especially for microplastics fractions lower than 20 µm. It is based on inelastic scattering of monochromatic light from a laser source by molecular bond vibrations. If combined with microscopy (micro-Raman), this technique enables the spectra analysis of polymer particles of few µm [20]. Furthermore, using Raman scattering with confocal laser microscopy, it is possible to detect microplastics even in biological tissues [17]. The main drawback of the Raman techniques is that the identification accuracy can be negatively affected by the presence of fluorescent pigments or additives in microplastics, which alter the vibrational information.

Spectroscopic techniques are considered the most reliable techniques for the identification of microplastics, but require a lot of time for analysis and complex and expensive equipment, not suitable for on-field analysis.

**Figure 2:**
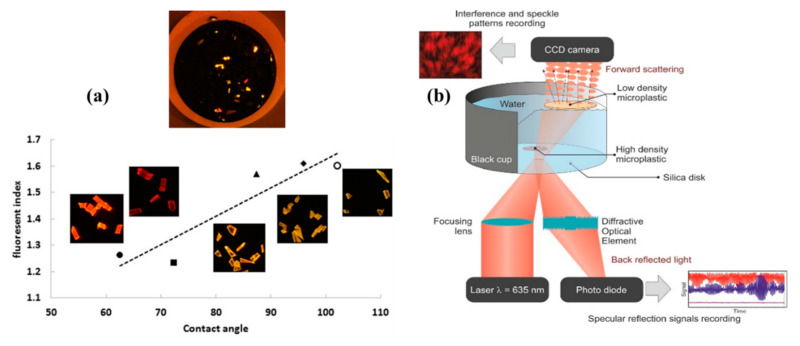
(**a**) Microplastics of six different polymer types dyed with Nile Red taken with a blue light (450−510 nm) and orange filter (529 nm) and their fluorescent index as a function of contact angle (reproduced from [21]); (**b**) schematic of a portable prototype optical sensor for detection of both transparent and translucent microplastics in water (reproduced with permission from [22]).

An alternative strategy for the identification and quantification of microplastics analysis was reported in [21], consisting of a fluorescence staining method in combination with density separation. Sediments samples are treated with a fluorescent dye known as Nile Red, which causes microplastics to brightly fluoresce when irradiated with blue light and enables them to be differentiated from surrounding particles (Figure 2a). Using a simple camera and an orange filter, the authors detected particles down to a few micrometers. Furthermore, the solvatochromic nature of Nile Red allows the microplastics’ classification on the base of hydrophobicity of identified particles.

Notably, in another work, Asamoah et al. [22] developed a portable prototype optical sensor (Figure 2b) to reveal the presence of both transparent and translucent microplastics in water (polyethylene terephthalate (PET) and low density polyethyleneor (LDPE)). Combining two detection modes on the specular reflection signal and the transmitted interference pattern from the light microplastics in water, it is possible to distinguish the type and size of microplastics in a volume of freshwater. The transparent polyethylene terephthalate showed a higher specular signal than the translucent LPDE, which is conversely responsible for a distorted interference pattern.

So far, however, we miss broadly accepted portable techniques for monitoring the threat of microplastics, and this is a key area for future development.

## 4. Heavy Metals

Heavy metals pollutants including cadmium (Cd), chromium (Cr), zinc (Zn), mercury (Hg), lead (Pb), arsenic (As), silver (Ag), copper (Cu), iron (Fe), and platinum (Pt) represent another serious environmental risk when present either in the more toxic free form (labile complexes, hydrated cations) or in stable complexes (with natural organic binders). They can be released naturally or by anthropogenic sources and their toxicity frequently arises through exchange and co-ordination mechanisms with proteins and enzymes then forming stable bio toxic compounds [23]. Heavy metal disorders and symptoms mainly arise following their transfer to the food chain because, when they come in contact with the soil, they tend to form minerals assimilated by the plants [24]; high concentrations of heavy metals were associated with neurological disorders, cancer, liver damages, diseases of the cardiovascular system, and hypophosphatemia, to name a few [25].

**Figure 3:**
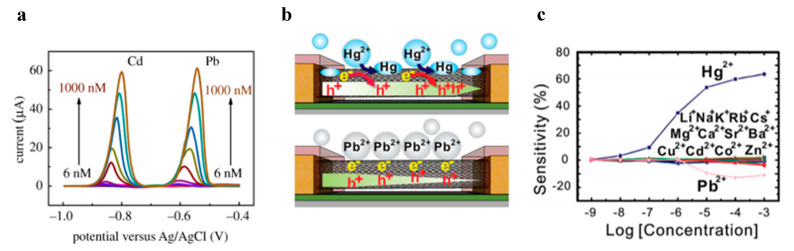
Heavy metal ions detection methods based on differential pulse stripping voltammetry DP-ASV (**a**) and conductance measurements from single−walled carbon nanotube field effect transistor (swCNT−FET) (**b**,**c**). In more detail, (**a**) calibration curves of the stripping peak currents at carbon black−Nafion−glassy carbon electrode with increased concentrations of Cd(II) and Pb(II) (reproduced from [31]). (**b**) Mechanism of detection for Hg^2+^ (upper) and Pb^2+^ (lower) by a swCNT−based FET sensor. (**c**) Response to various metal ions with concentrations from 1 nM to 1 mM (reproduced with permission from [36]).

To determine the presence of heavy metals in solutions, electrochemical methods and in particular anodic stripping voltammetry are often employed owing to their high sensitivity and ability to distinguish between labile and non-labile species/complexes. Moreover, electrochemical sensors are usually compact and can be miniaturized for on-field analysis. By anodic stripping voltammetry, it is possible to exploit the specific redox potential associated with each heavy metal ion for their assessment (identification) without a molecular recognition probe, while the anodic peak current value also allows their quantification [26]. In this respect, nanostructured/modified electrodes represent a new approach, where, for example, the employment of nanoparticles (NPs)-modified electrodes was shown to improve the selectivity, especially for As^3+^ and Hg^2+^ detection [27,28], thus eliminating the memory effect. On the other side, carbon nanomaterials can also ensure excellent electron transferability. In this way, the lowest limits of detection reach 0.04 ppb for Pb^2+^, 0.02 ppb for Cd^2+^, and 15 ppb for Cu^2+^ [29,30]; typical curves on the evaluation of heavy metal ions concentrations through nanostructured carbon black are reported in Figure 3a, based on differential pulse anodic stripping voltammetry (DP-ASV) [31]. In addition, the reduction of electrode size obviates problems such as a high potential for analyte deposition, high stripping potential of the analyte, and low selectivity. Micro- and nano-electrodes arrays were used, for example, to evaluate Cd^2+^ with a sensitivity until 40 ng/L in aqueous solution [32]. Microfluidics provides further valid support for long-term detection and setup miniaturization [33].

Remaining among electrical transduction approaches, field effect transistors (FETs) represent an emerging field for heavy metals detection using nanowires, carbon-based nanotubes, graphene [34], and NPs because of their high sensitivity, further emphasized with the employment of 1D and 2D nanomaterials [35]. FETs are sensitive to concentrations of Hg^2+^ and Cd^2+^ of 10^-7^ M and 10^-4^ M, respectively. Hg^2+^ presence was also evaluated by realizing FETs with single-walled carbon nanotubes [36], whose mechanism of detection is reported in Figure 3b; the device shows lower sensitivity with respect to Si-nanowire gated FET, but a higher detection range, as illustrated in Figure 3c. However, the highest sensitivity was reached recently by functionalizing graphene oxide on an FET, then obtaining a limit of detection as low as 2.5 x 10^-8^ M for Hg^2+^ ions in drinking water [37,38].

Optical transduction enables further approaches. For example, colorimetric detection of Hg^2+^ and Pb^2+^ were reported in [39] and [40] with limit of detection down to ten ppb for Hg^2+^. Chemiluminescence enables to reduce this threshold by a factor of 10 [41]. Fluorescent sensors represent versatile tools for the evaluation of the presence of heavy metals based on analyte-induced changes in the physicochemical properties of fluorophores. In this respect, beyond traditional organic dyes, emerging fluorophores include inorganic quantum dots (QDs) [42] and metallic nanoparticles [43], which are suitable for both *in vitro* and *in vivo* detection of heavy metals. In fluorescence sensing, Pb^2+^ ions were detected either by designing a QD–aptamer–graphene oxide (GO) sensor [44] or using DNAzyme–GO structures; the aptamer-quenching or QD-quenching of the fluorescence in the QD–aptamer–GO frame leads to evaluating the presence of Ag^+^ ions, beside Hg^2+^, reaching a limit of detection of almost 1 nM in aqueous solution [45]. In a different work, Cu^2+^ and Pb^2+^ concentrations were monitored by exploiting the surface plasmon resonance of Au or Ag NPs on a fluorescent sensor, giving a sensitivity of 2 ppt [46,47]. The presence of Hg^2+^, Pb^2+^, Cu^2+^, Cd^2+^, Mn^2+^, and other ions in aqueous solutions was also evaluated by employing plasmonic sensors based on nucleotide-functionalized Au NPs, both by colorimetric assessment and transmission localized surface plasmon resonance (SPR) spectroscopy [39,40,46,48].

The direct detection of heavy metal oxide groups was extensively carried out by exploiting surface enhanced Raman scattering (SERS) [49], especially for actinides [50], VI B–VII B group ions [51], and As^3+^ [52]. For this purpose, plasmonic nanostructures were functionalized with an organic ligand that binds specifically to heavy ions. An example of the structures that come to be formed is the self-assembled nanostar dimer based on the thymine-thymine pair of ssDNA, mediated by metal ion [53]; through this structure, it was possible to achieve a limit of detection of 0.8 pg/mL with a linear range from 0.002 to 1 ng/mL. The integration of the aforementioned sensors with high selectivity in microfluidic layouts gives an opportunity to realize optofluidic sensors, which enables real-time detection of multiple analytes [54].

## 5. Combustion Products and Hazardous Gases

Combustion products and hazardous gases, such as CO, CO_2_, NO_x_, SO_2_, and volatile organic compounds, are relevant threats for human health, severely affecting air quality and airways. Carbon monoxide, CO, causes poisoning, and is generally a product of poorly combusted organic materials and fossil fuels. Carbon dioxide, CO_2_, is present in air at relatively higher concentrations and is used also for human purposes (e.g., extinguishers, carbonating drinks), but it is also a product of fuels combustion, such as coal, methane, and petrol, and can be dangerous for the environment because it absorbs infrared photons, producing the well-known greenhouse effect, jointly with other gases [55]. Other molecules of interest for environmental monitoring are NO_x_, mainly coming from the combustion of fossil fuels in engines and industrial processes. NO_2_ is toxic, leading to health issues, especially related to the lungs. Sulfur dioxide, SO_2_, mainly comes from industrial activity [55] and can cause irritations in airways; acid rain is also a consequence of the presence of this gas in the environment. Moreover, volatile organic compounds (VOCs) are also indoor pollutants; some examples are propanol, toluene, ethanol, acetone, and so on. These are organic chemicals presenting a high vapor pressure at ordinary room temperature, and hence they can be dispersed in the air in a certain concentration. Owing to the increasing pollution in residential and industrial areas, there is an increasing need for technologies to monitor hazardous gases in the environment. Key factors in these investigations are portability, reusability, reliability, low cost, scalability, and real-time detection.

**Figure 4:**
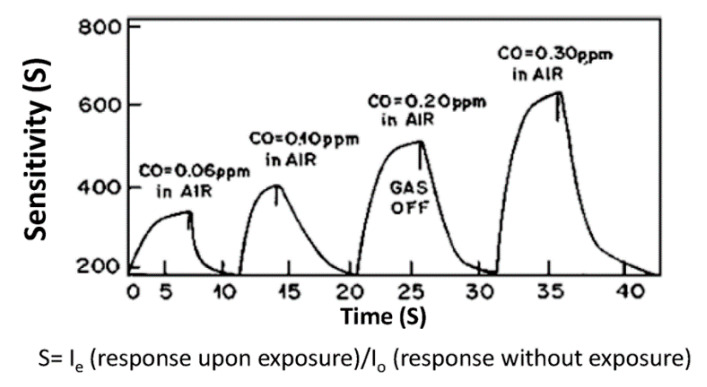
Response of a sensor based on a PANI−Fe:Al (80:20) nanocomposite thin film for CO in the range of 0.06−0.3 ppm (reproduced with permission from [58]).

During the years, various detection methods have been developed. In this respect, metal oxides-based gas sensors were widely investigated in the first 2000s decade [56], but a major limiting factor is the requirement of a pre-heating phase to 100–400 °C, which also makes their applications for explosive gas detection difficult. The sensing mechanism, in fact, is based on the adsorption/desorption of O ions (e.g., O^-^ and O^2-^), which is favored by the heating phase and makes the material responsive to the gas analytes [55], resulting in changes of charge distribution on the material’s surface, and thus a measurable variation of its conductivity. Owing to these limitations, a wide range of novel active-layer materials have been developed. Among them, to date, nanostructured hybrid materials appear to be among the best solutions, owing to their tunability and excellent sensing performances [57]. For example, vacuum-deposited PANI-Fe:Al (80:20) nanocomposite thin films were employed as active layer in sensors for rapid and selective detection of CO at ppb level, in the range of 0.06 to 0.3 ppm at room temperature [58] (Figure 4), with response times of the order of 10 s and good performance in terms of reusability.

## 6. Pesticides/Biocides

Another important class of hazardous contaminants concerns pesticides or biocides as chemical substances widely employed in agriculture to defend the crop from proliferation of undesirable biological organisms such as pest, insect, and weeds. Although the use of pesticides plays a significant role in the enhancement of agricultural productivity, their residues can cause soil, water, and air pollution (moving from one ecosystem to another), and even enter into the food chain. Maximum residue limits have been established by regulators, however, their bioaccumulation and the continuous exposure to them continue to have a negative impact on the environment and human health.

Among pesticides/biocides, those characterized by a high mortality rate are organochlorines (OCs), herbicides, organophosphates (OPs), fungicides and carbamates, and polychlorinated biphenyls (PCBs) [59] in general. In particular, the highest percentage of fatality is recorded following the employment of paraquat, fenthion, endosulfan, dimethoate, carbosulfan, and propanil [60]. The effects on human health after the assumption of contaminated drinking water or food range from acute poisoning (at the expense of the digestive and cardiovascular systems) to chronic intoxication [59] like acute respiratory distress syndrome (ARDS) with pulmonary fibrosis. Patients may also develop other life-threatening complications, such as liver dysfunction, acute tubular necrosis, and kidney failure [60]. Even the microorganisms useful for the aquatic ecosystems are heavily affected by the biocides, as reported in [61]. For all these reasons, the detection of pesticides is a crucial request to guarantee food safety and quality, protect the ecosystems, and safeguard human health from possible hazard.

Traditionally, chromatographic techniques such as liquid chromatography (LC), gas chromatography (GC), or capillary electrophoresis (CE) analysis have been used for pesticides analysis. Although these methods are sensitive and possess high specificity, they suffer from some drawbacks, such as time consuming procedures, high costs, sophisticated equipment that require skilled personnel, and a laborious sample preparation that limits on-site and on-field application. Therefore, research focused on the investigation of sensor technologies as an alternative to the standard analytic techniques for determination of various pesticides (organophosphates, organocloride, carbamates, and so on) in an easier, faster, low-cost, and user-friendly manner.

In the literature, several transduction mechanisms have been examined for pesticide detection using different recognition elements, such as enzyme, antibody, aptamer, or molecular imprinted polymers, as well as integrating nanomaterials to achieve higher sensitivity and selectivity. The most common tools in this field are enzyme-mediated sensors, in which the pesticides can act as inhibitor or as substrate for the enzymatic activity and detection is based on either indirect measurements of enzyme inhibition or direct measurements of substances involved in the enzymatic reaction.

Organophosphate and carbamate are common insecticides typically detected measuring the inhibition of acetylcholinesterase (AChE), an important enzyme for the functioning of the central nervous system. AChE hydrolyzes acetylcholine (ACh) to choline and acetic acid. Some pesticides are able to covalently bind at AChE active sites much more easily than ACh, inhibiting the enzyme activity. The activity tests can be carried out measuring reactants and products variations by means of different kinds of transducers (optical, electrochemical, piezoelectric). Fluorescence-based sensors are the most commonly applied for field-use pesticide monitoring because the signal change can be recorded through a portable spectrophotometer or visible by naked eye on site. For example, in [62], the authors developed a novel biosensor for the detection in a real sample of organophosphorus pesticides, based on the quenching of fluorescence of CdTe quantum dots in the presence of H_2_O_2_. The pesticides’ concentration can be also estimated by monitoring pH changes produced by the acid formation in the enzymatic reaction using potentiometric sensors [63]. In [64], the authors developed a portable amperometric biosensor for the rapid on-site detection of chlorpyrifos pesticides residues in fruits and vegetables with a low detection limit 100 ng/L and a measurement time of 15 min. Enzymatic-based biosensors are useful tools for rapid pesticide detection, but can suffer for a lack of selectivity as other compounds like heavy metals, fluoride or nicotine can also inhibit the enzymes.

Immunosensors can be considered a valid alternative to enzyme-based devices as they are able to distinguish different kinds of pesticides, being specific for a particular chemical moiety. In particular, they are based on the detection of specific antigen–antibody interactions by a transducer (electrochemical, optical, piezoelectric, and so on), which converts the biosensing event into a readable form. Numerous studies were carried out by means of quartz crystal microbalance immunosensors (QCM) in sample water for detecting various pesticides, such as the well-known gliphosate (LOD from 4 µg/L to 250 µg/L [65]). In [66], the authors developed a rapid immunochromotographic test strip based on Ab-Ag strategy for organophosphorus pesticide metabolite (TCP) investigation with a limit of detection of 1.0 ng/mL and a response time of 15 min. A high sensitive SPR immunosensor was also reported by Guo et al. for monitoring thiazophos in agricultural crops and water, with an LOD of 0.096 ng/mL and a linear range of 0.98–8.29 ng/mL [67]. The main drawback of the use of immunosensors for detection of pesticides is the long and expensive production of specific antibodies for these very small and highly toxic molecules.

**Figure 5:**
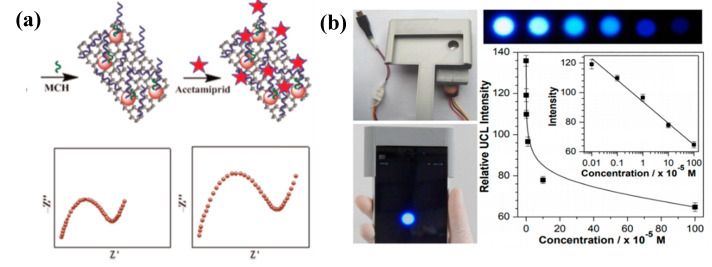
(**a**) Schematic representation of impedimetric aptasensor for detection of acedamiprid (reproduced with permission from [70]). (**b**) Image of smartphone−based detection setup and of luminescence test paper, and upconversion luminescence (UCL) spectra upon additions of different amounts of thiram (reproduced with permission from [71]).

A good alternative as recognition elements in the development of biosensors for pesticide residues detection is represented by aptamers for their several advantages over antibodies such as high specificity, low molecular weight, wide range of targets, easy synthesis, and modification. Aptamers are single-stranded RNA or DNA sequences synthetized for selectively binding to target molecules with high affinity. The aptamer molecules are used as receptors similarly to antibodies in sensors with different transducer mechanisms (colorimetric, electrochemical, fluorescence, SERS). Colorimetric aptasensors are widely applied for the detection in real time of pesticides pollutants in environment and food for easy sample preparation and the possibility to observe the results with naked eyes. For example, in [68], the authors developed a colorimetric aptamer assay for the detection of organophosphorous omethoate, using the resistance of single-stranded DNA-wrapped Au NPs against salt-induced aggregation. In the presence of pesticide, the aptamer binds to the omethoate, separating from gold nanoparticles, which aggregate, resulting in a color solution change. This aptasensor exhibited a good linearity between 0.1 and 10 μM and a low detection limit of 0.1 μM. In [69], a double-stranded DNA was employed to prevent Au NP aggregation in salt solution for colorimetric detection of pesticide malathion. In this case, a linear range from 5 pM to 10 nM and lower detection limit of 1 pM were achieved. For its high sensitivity and low cost, electrochemical impedance spectroscopy is the most commonly used strategy in the development of electrochemical aptasensors. In [70], an ultrasensitive EIS aptasensor was built up by Fei et al. to detect residues of insecticide acetamiprid at femtomole level (LOD of 17 fM), employing gold nanoparticles (Au NPs) decorated multiwalled carbon nanotube-reduced graphene oxide nanoribbon composites as support for aptamer immobilization (Figure 5a). Molecular imprinted polymers can be another valuable alternative to antibodies and aptamers as specific molecular probes.

Concerning fluorescence transduction mechanism, a smartphone-based prototype system (Figure 5b) was developed for real-time detections of the highly toxic pesticide thiram on NaYF4:Yb/Tm up-conversion nanoparticles modified test paper. The luminescence variation of nanoparticles fixed onto filter paper is related to the amounts of thiram deposited on the surface and was monitored by a smartphone camera [71]. In [72], the authors realized flexible and controllable paper-based SERS (surface enhanced Raman scattering) swabs with silver nanoparticles (Ag NPs) and graphene oxide (GO) using the screen-printing technique for the determination of pesticide residues in fruits and vegetables. This sensing platform was able to detect different types of pesticides such as thiram, thiabendazole, and methylparathion, with detection limits of 0.26 ng/cm^2^, 28 ng/cm^2^, and 7.4 ng/cm^2^, respectively.

## 7. Noise

Beyond being annoying, unwanted acoustic noise can also psychologically and physiologically impact health [73], from hearing loss to lack of cognitive performances, sleep disturbance, up to cardiovascular diseases [74]. In terms of regulation, World Health Organization (WHO) defines periodically recommendations about limit values, regarding noise peaks and the so-called *Day-Evening-Night-Level* (L_DEN_), which represents a measurement of acoustic intensity averaged in a whole day, with “penalties” of 10 dB during the night and 5 dB during the evening. In the 2018 updated version for Europe [75], WHO suggested to maintain $L_{DEN}<54\;\mathrm{dB}$ for road traffic noise, $L_{DEN}<53\;\mathrm{dB}$ near railways, and $L_{DEN}<45\;\mathrm{dB}$ in airports. The first step to assess noise level is the evaluation of the acoustic pressure in decibels (dB), with 20 μPa as reference value being the average human limit of detection for a 1 kHz sound [73], but the sound frequency has to be specified because of the changing sensitivity within the human’s hearing range (20 Hz–20 kHz, with a maximum at few kHz) [76]. For this reason, the IEC61672-1:2002 standard suggests to normalize noise measurements at the levels really perceived by the human’s auditory system by means of weighted filters [76].

The golden standard in acoustic noise assessment is the employment of professional sound level meters (SLMs), which are continuously recording devices suitably positioned in the environment for on-field measurements of the acoustic spectrum [77]. As disadvantages, they are not very user-friendly and cheap enough for an average worker to buy and use. For this reason, Nast et al. in 2014 [78] proposed individual sound level measurements by free smartphones applications. Their comparative study showed that this technology was not as accurate as type 2 SLMs [78], but it is possible to improve the performances through advanced calibration procedures and non-linearity correction, approaching the professional SLM level. This procedure, however, must be performed by an expert, again making it not possible for anyone to perform a noise self-assessment. Three years later, Zamora et al. [79] performed a systematic study about the feasibility of mapping the noise inside an area through a distributed noise sensing units consisting of different smartphone platforms by optimizing several parameters from the algorithm to sampling and data gathering processes, finally obtaining performances comparable to the employment of professional SLM devices, but the need to take into account the different characteristics of smartphones coming from different vendors, the aptitude of low-end devices to introduce errors more pronounced than high-end ones, and the difficulty to measure the same space point for long time are features that complicate the application of this *crowdsending* approach in noise pollution monitoring. Regarding indoor measurements, for example, in factories, the actual trend is compliant with the present Internet Of Things philosophy employing a series of low cost distributed sensors and integrating microcontroller units that gather the data from the sensing expansion, perform a first manipulation (e.g., filtering), and send data to a cloud archive for further heavier calculations. For example, in 2018, Risojević et al. [80] optimized the performances of limited computational resources and cheap devices (any node costs about 41 €) to obtain a system able to monitor the environmental noise for several days with a precision similar to that reachable with professional sound meter devices. Recent advances have led to the implementation of this approach not only to map the noise pollution in outdoor environment, but also to gather sound data that can be analyzed by a server through convolutional neural networks in order to classify them by individuating their source, as well as allowing the user able to receive and visualize noise maps, acoustic events information, and noise statistics in a defined area [81].

## 8. Discussion

Significant progresses have been achieved in the design and implementation of portable sensors for environmental monitoring. Today, particulate matter, microplastics, heavy metals, small molecules as combustion products, and hazardous gases in air or pesticides/biocides in water can be detected with unprecedented capability. For each class, Table 1 summarizes the current guideline values, health effects, sources, and achieved limit of detection, also mentioning the employed transduction approaches in order to guide researchers, stakeholders, and regulators to define their strategies and policies. It should be specified that the thresholds beyond which a product is considered harmful have not been established for all the contaminants/pollutants reported in Table 1, as well as reliable detection methods have not yet been established for each pollutant. Moreover, although the availability of novel (nano)materials and fabrication capabilities created new opportunities for improving the figures of merits, there are no ideal sensors for all scopes, but the technology of choice depends on the specific application, the measurement/environmental constraints, the need for portability and field use, as well as a compromise among costs and sensitivity requirements.

To further increase the impact of environmental sensor technologies, their combination with robotics and Internet of Things (IoT) can be exploited to achieve efficient, unmanned monitoring at unprecedented scales, in conditions of particular interest such as after natural disasters and environmental accidents, but also in unstructured environments, like damaged nuclear power plants [87], active volcanoes [88], and deep oceans [89]. In particular, useful emerging trends in robotics range from the establishment of cooperative robotic teams, to the improvement of the interaction between robots and a wireless sensor network (WSN), from the planning of model-aided paths to adaptive sampling. The realization of a group of vehicles equipped with sensor suites, moving collectively and cooperatively, can pave the way to a revolution, because of its dynamicity, flexibility, and suitability for multiple source localization, even if this approach requires fixing many technical issues related to endurance, planning, coordination, communication, and cooperation. An example of cooperative systems is reported in [90], while a review of the various approaches in this direction is presented in [91]. On the other hand, a WSN composed of autonomous sensors is capable of measuring a number of environmental parameters and of locally processing and storing the acquired data. While WSNs are perfect in monitoring the environment, they are very limited in reacting to what they detect. In this respect, robots can act as interfaces to WSN, then enhancing them by providing important benefits such as sensor deployment, calibration, failure detection, and power management. The requirements characterizing the interaction among robots and WSN and how these elements are being addressed in the design of the new communication framework are discussed in [92]. For what concerns the model-aided path planning in robotics, its integration with forecast models of environmental parameters allows to improve performance like orientation and endurance in dynamics environments; the approach is based on the realization of early scientific environmental models, the examples of which are reported in [93,94,95]. Furthermore, adaptive sampling represents another solution when physical phenomena show unknown spatial distribution that also changes over time; in these cases, it is possible to use multiscale algorithms for characterizing particular features, as reported in [96].

Similarly, the development of the Internet of Things gives the possibility to deploy sensor networks that acquire data in real time from sensor nodes in widely distributed areas. An intriguing perspective can be represented by the Internet of Nano-things [97], which is achieved by incorporating nano-sensors into many objects and by using nano-networks [98]. This recent conception pushes technology even further towards the realization of wearable nano-sensors in spite of many current sensors, which do not give the possibility to wear them because of their dimensions and the rigid materials of which they are composed. In this direction, the convergence between nanobiotechnology and Internet of Things (IoT) can be exploited for the implementation of monitoring systems and the optimization of environmental conditions in plants, as well as with the use of UAVs (unmanned aerial vehicles) and with ground stations. The requirements that nano-sensors must meet in view of their use with IoT and drones are mainly the reliability in highly variable environmental conditions, simplicity in replacing the in-field sensors, longevity, and the ability to communicate data. Given the assumptions, the promise of using these new technologies for both air and drinking water monitoring can be maintained within the next few years.

## 9. Conclusions

In conclusion, a wide ensemble of different pollutants threatens the environment and human health. Their various forms set specific requirements for monitoring tools and campaigns. However, thanks to significant progresses in sensor platforms, we now have available unprecedented technologies and building blocks that are expected to enable breakthroughs for next-generation environmental monitoring.

## Figures and Tables

**Table 1:** Summary of contaminants characteristics, effects, and available sensing technologies for their monitoring.

*Pollutant*	Guideline Value	Health Effects	Sources	Limit of Detection
***PARTICULATE MATTER (PM) [5]***	**PM**_2.5_: 25 µg/m^3^ (1 d). **PM**_10_: 50 µg/m^3^ (1 d).	Acute lower respiratory infections, cardiovascular disease, chronic obstructive pulmonary disease, and lung cancer.	Mainly in developing cities, in particular in South East Asia and countries in Western Pacific Ocean.	**PM with 0.3 um minimum dimension**: 1 µg/m^3^ [*light-scattering photometry*].
***MICROPLASTICS [82,83]*** ***polyethylene (PE), polypropylene (PP), polyvinyl chloride (PVC), polystyrene (PS), polyurethane (PUR), polyethylene terephthalate (PET)***	*Not yet established*	Irritation on eyes, respiratory tract symptoms, liver and gastrointestinal effects, neurobehavioral and immunological changes in children, miscarriage, damage to immune system, endocrine disruption, decreased comprehension.	Bags, storage containers, bottles, gear, strapping, cool boxes, floats, cups, utensils, film, pipe, fishing nets, rope, boats, cigarette filters.	*Not yet established*
***HEAVY METALS*** *[84,85]* ***Sb, As, Cd, Cu, Pb, Se, Ag, U, Hg, Fe, Cr, Zn***	**Sb**: 0.02 mg/L; **As**: 0.01 mg/L; **Cd**: 3 µg/L; **Cu**: 2 mg/L; **Cr**: 0.05 mg/L; PDMI of Fe: 0.8 mg/kg; **Pb**: 0.01 µg/L; **Hg**: 6 µg/L; **Ni**: 0.07 mg/L; **U**: 0.03 mg/L.	Hyper-pigmentation, hypo-pigmentation, neuropathy, skin and lung cancer, gastrointestinal disturbances, hypertension, impaired fertility, tubular necrosis, proteinuria, hypoalbuminaemia, gastritis haemorragic, argyria, nephritis.	Corrosion of pipes and steel during water distribution, lubricant agents in petrol, lead-acid batteries, steel industries and alloys industries, fertilizers, granites, and nuclear power stations.	**Sb**: 0.01 µg/L [*AAS*]; **Cr**: 0.05 µg/L [*AAS*]; **Pb**: 1 µg/L [*AAS*]; **Hg**: 0.05 µg/L [*AAS*]; **Se**: 0.5 µg/L [*AAS*]; **As**: 0.1 mg/L [*ICP-MS*]; **Cd**: 0.01 µg/L [*ICP-MS*]; **Cu**: 0.02 µg/L [*ICP-MS*]; **Ni**: 0.1 µg/L [*ICP-MS*]; **U**: 0.01 µg/L [*ICP-MS*].
***COMBUSTION-PRODUCTS** [86]* ***O_3_, NO_2_, SO_2_***	**O_3_**: 100 µg/m^3^ (8-h) **NO_2_**: 40 µg/m^3^ (1-y) **SO_2_**: 20 µg/m^3^ (1-d)	Inflammation of airways, asthma, chronic obstructive pulmonary disease, reduced lung function, proclivity to infection of the respiratory tract.	Photochemical smog, reaction between NOx and VOCs from vehicles, solvents and industry, burning of fossils fuels, smelting of mineral ores.	**SO_2_**: 0.1 ppm [*EC*]; **O_3_**: 0.01 ppm [*EC*]; **NO_2_**: 0.1 ppm [*EC*].
***HAZARDOUS GASES/HYDROCARBONS*** [85] ***Acrylamide, brominated acetic acid, Carbon tetrachloride, Chloral hydrate, Chloramines, 2,4,6-Trichlorophenol, Dialkyltins, 1,2-Dibromoethane, Dichloroacetic acid, 1,2-Dichloroethane, 1,2-Dichloroethene, Dichloromethane, 1,2-Dichloropropene, Di(2-ethylhexyl)phthalate, 1,4-Dioxane, Edetic acid, Epichlorohydrin, Formaldehyde, MTBE, PAHs, Styrene, Tetrachloroethene, Vinyl chloride.***	**Acrylamide**: 0.5 µg/L; **Carbon tetrachloride**: 4 µg/L; **Chloramines**: 3 mg/L; **1,2-Dibromoethane**: 0.4 µg/L; **Dichloroacetic acid**: 50 µg/L; **1,2-Dichloroethane**: 30µg/L; **1,2-Dichloroethene**: 50 µg/L; **Dichloromethane**: 20 µg/L; **1,2-Dichloropropene**: 20 µg/L; **Di(2-ethylhexyl)phthalate**: 8 µg/L; **1,4-Dioxane**: 50 µg/L; **Edetic acid**: 0.6 mg/L; **PAHs**: 0.7 µg/L; **Styrene**: 20 µg/L; **Tetrachloroethene**: 20 µg/L; **Vinyl chloride**: 0.3 µg/L.	Neurotoxicity, affection of germ cells, impairment of reproductive functions, scrotal, thyroid, and adrenal tumors, oral toxicity, hepatomas, hepatocellular carcinomas, mononuclear cell leukaemia, forestomach tumor, nasal cavity tumor, increase of serum glutamate-pyruvate transaminase level, central nervous system depression, angiosarcoma, liver cancer.	Treatment of drinking water, production of plastics, resins and other organic chemicals, civil use and industrial materials treatment.	**Acrylamide**: 32 ng/L [*GC*]; **Carbon tetrachloride**: 0.1 µg/L [*GC-ECD/MS*]; **Chloramines**: 10 µg/L [*Col*]; **Dialkyltins**: 0.01 µg/L [*GC-MS*]; **1,2- 1,2-Dichloroethane**: 0.1 µg/L [*GC-ECD*]; **1,2-Dichloroethene**: 0.17 µg/L [*GC-MS*]; **Dichloromethane**: 0.3 µg/L [*GC-MS*]; **1,2-Dichloropropene**: 0.2 µg/L [*GC-ECD*]; **Di(2-ethylhexyl) phthalate**: 0.1 µg/L [*GC-MS*]; **1,4-Dioxane**: 0.1 µg/L [*GC-MS*]; **Edetic acid**: 1 µg/L [**potenziometric stripping**]; **Epichlorohydrin**: 0.01 µg/L [*GC-ECD*]; **PAHs**: 10 ng/L [*GC-MS*]; **Styrene**: 0.3 µg/L [*GC/PID-MS*]; **Tetrachloroethene**: 0.2 µg/L [*GC-ECD*]; **Vinyl chloride**: 10 ng/L [*GC-ECD*].
***PESTICIDES** [84]* ***alachlor, aldicarb, aldrin, dieldrin, atrazine, bentazone, carbaryl, carbofuran, chlordane, chlorotoluron, chloropyrifos, cyanazine, 2,4-D, 2,4-DB, DDT, 1,2-dichloropropane, dichlorprop, dichlorvos, dicofol, dimethoate, diquat, endosulfan, entrin, fenitrothion, fenoprop, glyphosate, isoproturon, lindane, malathion, MCPA, mecoprop, methoxychlor, methylparathion, metolachlor, molinate, parathion, pendimethalin, pentachlorophenol, propanil, simazine, 2,4,5-T, terbuthylazine, trifluralin***	**Alachlor**: 0.02 mg/L; **aldicarb**:0.01 mg/L; **aldrin, dieldrin**:0.03 µg/L; **atrazine**:0.1 mg/L; **bentazone**:0.5 mg/L; **carbaryl**:50 µg/L; **carbofuran**:7 µg/L; **chlordane**:0.2 µg/L; **chlorotoluron**:30 µg/L; **chloropyrifos**:30 µg/L; **cyanazine**:0.6 µg/L, **2,4-D**:30 µg/L, **2,4-DB**:90 µg/L; **DDT**:1 µg/L; **1,2-DCP**:20 µg/L; **dichlorprop**:100 µg/L; **dichlorvos**:20 µg/L; **dicofol**:10 µg/L; **dimethoate**:6 µg/L; **diquat**:30 µg/L; **endosulfan**: 20 µg/L; **entrin**:0.6 µg/L; **fenitrothion**:8 µg/L; **fenoprop**:9 mg/L; **glyphosate**:0.9 mg/L; **isoproturon**:9 µg/L; **lindane**:2 µg/L; **MCPA**:0.7 mg/L; **mecoprop**:0.01 mg/L; **methoxychlor**:0.02 mg/L; **metolachlor**:0.01 mg/L; **molinate**:6 µg/L; **parathion**:10 µg/L; **pendimethalin**:20 µg/L; **pentachlorophenol**:9 µg/L; **simazine**:2 µg/L, **2,4,5-T**:9 µg/L; **terbuthylazine**:7 µg/L; **trifluralin**:20 µg/L.	Turbinate, stomach, thyroid cancer, inhibition of acetylcholinesterase, liver tumor, destruction of estrous cycle, kidney toxicity, inhibition of brain acetylcholinesterase, soft tissue sarcoma, non-Hodgkin lymphoma, mitogenic effects, neurotoxicity, skin irritation, anaemia, hyperglycaemia.	Agriculture, urban pest control.	**Alachlor**: 0.1 µg/L [*G(L)C*]; **aldicarb**: 1 µg/L [*HPLC-FD*]; **aldrin**: 0.003 µg/L [*GC-ECD*]; **dieldrin**: 0.002 µg/L [*GC-ECD*]; **atrazine**: 5 ng/L [*HPLC-UVPAD*]; **bentazone**: 0.01 µg/L [*LC-MS*]; **carbosulfan**: 0.1 µg/L [*HPLC-FD*]; **chlordane**: 0.014 µg/L [*GC-ECD*]; **chlorotoluron**: 0.1 µg/L [*HPLC-UVD*][*EC*]; **chloropyrifos**: 1 µg/L [*GC-ECD*]; **cyanazine**: 0.01 µg/L [*GC-MS*]; **2,4-D**: 0.1 µg/L [*G(L)C- ECD*]; **2,4-DB**: 1 µg/L [*HPLC-ECD (UVD)*]; **chlorodiphenyltrichloroethane**: 11 ng/L [*GC-ECD*]; **1,2-dichloropropane**: 20 ng/L [*GC-ECD*]; **1,3-dichloropropene**: 0.2 µg/L [*GC-ECD*]; **dichlorprop**: 1 µg/L [*HPLC-ECD (UVD)*]; **dichlorvos**: 10 ng/L [*GC*]; **dicofol**: 5 ng/L [*GC*]; **dimethoate**: **diquat**: 1 µg/L [*HPLC-UV*]; **entrin**: 2 ng/L [*GC-ECD*]; **fenoprop**: 0.2 µg/L [*GC-ECD*]; **isoproturon**: 0.1 µg/L [*ozonation*]; **lindane**: 0.01 µg/L GC; **MCPA**: 90 ng/L [*GC-ECD*]; **mecoprop**: 10 ng/L [*GC-ECD*]; **methoxychlor**: 1 ng/L [*GC*]; **metolachlor**: 0.01 µg/L [*HPLC-FD*]; **molinate**: 10 ng/L [*GC-MS*]; **parathion**: **pendimethalin**: 10 ng/L [*GC-MS*]; **pentachlorophenol**: 5 ng/L [*GC-ECD*]; **propanil: simazine**: 10 ng/L [*GC-MS*]; **2,4,5-T**: 20 ng/L [*GC-ECD*]; **terbuthylazine**: 0.1 µg/L [*HPLC-UVD*]; **trifluralin**: 50 ng/L [*GC- FD*].
*NOISE*	**L_DEN_**: 54 dB (traffic). **L_DEN_**: 53 dB (railways). **L_DEN_**: 45 dB (aircraft).	Lack of cognitive performances, sleep disturbance, cardiovascular disease, hearing loss.	Traffic, railways, aircrafts, factories’ instrumentation, concerts	**1 dB for type 1 sound level meters**

^1^ The acronyms for methods of analysis are [AAS]: atomic absorption spectroscopy; [ICP-MS]: inductively coupled plasma mass spectrometry; [HPLC-FD]: high-performance liquid chromatography-fluorescence detection; [HPLC-UVPAD]: high-performance liquid chromatography-ultraviolet photodiode array detection; [HPLC-ECD]: high-performance liquid chromatography-electron capture detection; [HPLC-UV]: high-performance liquid chromatography-ultraviolet detection; [GC]: gas chromatography (not specified); [Col]: colorimetric methods; [GC-MS]: gas chromatography/mass spectrometry; [GC-ECD]: gas chromatography–electron capture detection; [PID-MS]: photoinduced detection–mass spectroscopy; [EC]: electrochemical approach; [VOC]: volatile organic compound. (8 h), (1 d), (1 y): exposure times to various pollutants/contaminants, corresponding to 8 h, 1 day, and 1 year, respectively.
